# Is “earth” an animate thing? Cross-language and inter-age analyses of animacy word ratings in European Portuguese and British English young and older adults

**DOI:** 10.1371/journal.pone.0289755

**Published:** 2023-08-04

**Authors:** Sara B. Félix, Marie Poirier, Josefa N. S. Pandeirada

**Affiliations:** 1 William James Center for Research, Department of Education and Psychology, University of Aveiro, Aveiro, Portugal; 2 Department of Psychology, School of Health and Psychological Sciences, University of London, London, United Kingdom; The Psychology Research Center (CIPSI), University of Minho, PORTUGAL

## Abstract

Animacy plays an important role in cognition (e.g., memory and language). Across languages, a processing advantage for animate words (representing living beings), comparatively to inanimate words (i.e., non-living things), has been found mostly in young adults. Evidence in older adults, though, is still unclear, possibly due to the use of stimuli not properly characterised for this age group. Indeed, whereas several animacy word-rating studies already exist for young adults, these are non-existent for older adults. This work provides animacy ratings for 500 British English and 224 European Portuguese words, rated by young and older adults from the corresponding countries. The comparisons across languages and ages revealed a high interrater agreement. Nonetheless, the Portuguese samples provided higher mean ratings of animacy than the British samples. Also, the older adults assigned, on average, higher animacy ratings than the young adults. The Age X Language interaction was non-significant. These results suggest an inter-age and inter-language consistency in whether a word represents an animate or an inanimate thing, although with some differences, emphasising the need for age- and language-specific word rating data. The animacy ratings are available via OSF: https://osf.io/6xjyv/.

## Introduction

Animacy plays a special role in cognition. However, several conceptualisations of animacy have emerged [1], making its operationalisation somewhat challenging. For example, animacy has been considered a synonym of *humanness* (classifying items as human/non-human, although not always explicitly, [2]) and of *agency* (having self-propelled motion and goal-directed behavior or not, [3]). Likewise, animacy has been conceived as a dichotomic variable (animate/inanimate, [4]), as a trichotomy (animate/ambiguous/inanimate, [5]), and also as a continuous variable (ranging from “totally inanimate” to “totally animate”, [6]). Given this variability, VanArsdall and Blunt [1] examined which constructs predicted animacy word ratings. They concluded that the living/non-living dimension was one of the constructs that explained the most variance in animacy ratings. Therefore, herein, we will conceive animacy as a continuous variable and a synonym of *livingness* (living/non-living). Additionally, animacy appears to be a unique variable, as it does not seem to share much variance with other constructs. For example, only low correlations have been reported (at least in Portuguese and English, [7, 8]) between animacy ratings and other semantic variables, such as concreteness (Portuguese: *r* = -.16; English: *ρ* = .04) and imageability (Portuguese: *r* = -.14).

One example of the role played by animacy in cognition comes from the memory literature. Using regression analyses, Nairne et al. [9] reanalysed Rubin and Friendly’s [10] data relative to the predictors of free recall. The results revealed that, among several well-known predictor variables, animacy was one of the best predictors of free recall performance. Since this seminal work, this finding has been replicated, both in terms of predicting the recall of individual words [8, 11], as well as of wordlists [4]. Alongside such evidence, the *animacy effect* (better memory for animates than inanimates) has been found to be a robust phenomenon, replicated in several languages, with different types of stimuli, as well as with various memory tasks. For example, it has been obtained in free recall and recognition with words and pictures in French [5], cued recall with nonwords associated with animate/inanimate descriptors in English [12], and in incidental and intentional learning followed by an immediate or delayed recall in Portuguese [13].

Regarding perception and attention, animates seem to capture attention faster and for longer periods of time than inanimates [14–16]. In language, animacy plays a central role in sentence construction as, across languages, animates typically are the agents within phrases [17]. Also, in verbal fluency tasks people usually generate more words from animate categories (e.g., animals) than inanimate categories (e.g., transports [18]).

Despite the growing evidence of the role of animacy in cognition, this variable is rarely controlled for in research. Still, there are word-rating studies available in some languages: American and Canadian English [1, 4, 19, 20], European Portuguese [21], Serbian [20], Persian [22, 23], Japanese [23] and Croatian [24]. In most of these studies, participants rated words on a 7-point scale. Others have reported the classification of words into the animate/inanimate categories [4, 24]. In all of these studies, data were obtained from young adults, which raises questions about their suitability to be used with other age groups.

One example of this possible issue can be found in the literature on the mnemonic animacy effect in older adults, in which the results are still scarce and non-consensual. Some studies have reported the animacy effect among healthy older adults [25], but others have shown a reduced or even absent effect in this age group (e.g., [26]). However, in these studies, the manipulation of animacy relied on animate/inanimate classifications provided by young adult participants. Considering possible cohort effects, where, for example, young and older adults process word meaning differently [27–30], relying on norms obtained with young adults, introduces a possible item-selection problem.

This work aims to provide the first animacy word ratings obtained from older adults in two languages: European Portuguese and British English. Additionally, we collected ratings from British English young adults. We also relied on data previously obtained with Portuguese young adults [21] to conduct comparisons between age groups and languages and explore commonalities and differences in the animacy ratings. Words were rated through an online questionnaire using the typical 7-point rating scale [1]. The database is available through OSF [31].

## Method

### Participants

This study included young and older adults as participants. For the Portuguese and British samples of older adults, we considered participants aged over 60 years old and over 65 years old, respectively. Both these age-limits have been considered in the literature as defining older adults (e.g., [32, 33]). We opted to use a slightly younger age group for the sample of Portuguese older adults due to the unfeasibility of recruiting only participants over 65 to participate in an online study in said population (e.g., computer-usage skills are relatively low in older Portuguese participants; maintaining the online data-collection procedure was essential to ensure comparability across samples). Young adults were aged between 18 and 35 years old in both the Portuguese and the British samples. Table 1 provides the characterisation of the various samples of participants. Data from the European Portuguese young adults (ranging between 18 and 35 years) were retrieved from Félix et al. ([21], *N* = 152). All sample sizes ensured the collection of at least 20 ratings per word from each sample, as is usual in other studies [1].

**Table 1:** Characterisation of the samples, mean ratings per word, and average animacy ratings.

Samples	% Female/Male	Mean age (*SD*; Range)	Mean ratings/word (*SD*; Range)	Mean ratings (*SD*)
Portuguese Older Adults	67.8 / 32.2	68.6 (7.1; 60–96)	29.50 (3.21; 27–35)	3.68 (2.16)
Portuguese Young Adults	82.2 / 17.8	23.4 (4.3; 18–35)	76.68 (6.61; 58–97)	3.51 (2.27)
British Older Adults	50.2 / 49.8	71.5 (4.6; 65–85)	25.87 (2.20; 22–30)	3.97 (2.61)
British Young Adults	44.7 / 52.2*	27.8 (5.1; 18–35)	20.12 (0.33; 20–21)	3.80 (2.62)

*SD* = Standard Deviation.

*2.5% of the participants in this sample identified themselves as “other” and 0.6% preferred not to answer to this question.

### European Portuguese older adults

Participants were 118 European Portuguese older adults, recruited through e-mail, social networks, and Senior Universities. An additional 20 participants were excluded (17 were below 60 years old or did not provide age information, and three were non-Portuguese native speakers). A total of 38 participants aged between 60 and 65 years old composed this sample. The mean animacy ratings given by this sub-group of participants and the remaining (aged ≥ 65 years old) was not statistically different, *t*(116) = -0.16, *p* = .877. Data were collected between July and November of 2020.

### British English older adults

Participants were recruited via the Qualtrics Research Services, using the following pre-screeners: gender-equated sample, location (UK), first language (English), and age (≥ 65 years old). The sample included 207 English-speaking older adults who provided valid responses, according to Qualtrics Services, in exchange for £3.80-rewards. Data were collected in December of 2020.

### British English young adults

Participants were 161 young adults recruited from Testable (https://www.testable.org/), using the following pre-screeners: age (18–35 years old), first language (English), and location (UK). They received $2.00 for their participation. An additional eight participants were excluded from the analyses (six were not English native speakers, one rated all words with the values of 5 and 6, and another one had a technical problem leading to the collection of no ratings). This study was pre-registered (https://aspredicted.org/hb4na.pdf), and data were collected between November and December of 2022.

### Materials

The Portuguese samples rated the 224 words from Félix et al. [21], in which the selection of this pool of words is described. In said study, the young adults rated lists of 112 words, randomly selected from the total pool of words. The Portuguese older adults rated lists of 56 words each that were created beforehand. Words for these lists were randomly selected, with the only constraint that all lists contained a similar proportion of animate, inanimate, and ambiguous words based on previous ratings [21]. The lists of to-be-rated words were then divided into four groups. In both samples, the presentation order of the words within each of the four groups was randomly determined for each participant while also ensuring a balanced presentation of each type of word (animates, inanimates and ambiguous words) within each group.

The British participants rated 500 words. This pool of words was selected from the VanArsdall and Blunt’s study [1], comprising the same proportion of animate (41%), inanimate (46%), and ambiguous words (14%) as in the original study, and according to their animacy categorisation criterion. We avoided selecting words of similar meaning (e.g., VanArsdall and Blunt’s database contains both “fall” and “autumn”, we opted for the last; in “plane” vs. “airplane” we opted for the British corrected form of the last–“aeroplane”). Words were corrected to British spelling (e.g., mom/mum) or their British equivalent based on meaning (e.g., movie/film; airplane/aeroplane). Eight lists of words were created beforehand, each composed of 62 or 63 words depending on the list. Each list contained a similar proportion of animate, inanimate, and ambiguous words. Words were presented in four groups, formed in a random manner for each participant. Within each group, they were also presented in a random order. Each British participant rated one of these lists.

S1 File presents the characterisation of the European Portuguese and British English words on several variables.

### Procedure

**Fig 1:**
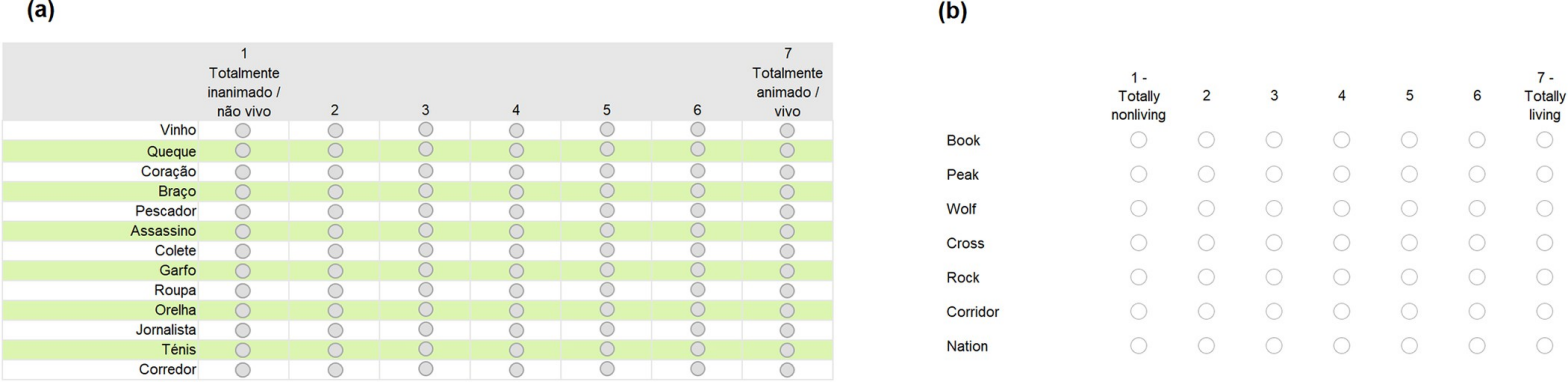
Example of the Animacy Rating Task in (a) European Portuguese and (b) British English.

We followed a procedure similar to that reported by VanArsdall and Blunt [1]. Generally, participants received the animacy rating instructions (see S2 File). Then, they were presented with four groups of words and rated them on a 7-point scale. Responses were mandatory for all items. Fig 1 depicts an example of one of those groups of words.

Following the best practices to ensure the quality of data collected online (e.g., [34, 35]), we have implemented two attention-check questions as used before [1, 21]. Specifically, after rating two of the groups of words, an attention-check was presented (e.g., “Have you ever walked on Mars?” Yes/No response). Then, a reminder of the instructions was presented, followed by the other two groups of words. Participants then responded to a new attention-check (e.g., “Can you fly with invisible wings?” Yes/No response). At the end of the survey, the young Portuguese and the British participants indicated if they paid attention to the study or not (as in [1], cf. [33]). Data were collected through LimeSurvey (Portuguese samples) and Qualtrics (British samples).

### Ethics statements

The procedures used to collect the data in the current study were positively appreciated by the Ethics and Deontology Committee of the University of Aveiro (for the older adult Portuguese sample; Ref: 34/2019) and the by City, University of London Psychology Research Ethics Committee (for the British samples; Refs: ETH1920-1021, ETH2223-0403) and are in accordance with the Declaration of Helsinki. All participants provided written informed consent prior to their participation. Participation was anonymous; therefore, we were unable to identify individual participants either during or after data collection. Given the aims of the study, young (aged between 18 and 35 years old) and older adults took part in this study. Given the aims of the study called upon, young (aged between 18 and 35 years old) and older adults (aged over 60 and 65 for the Portuguese and British samples, respectively) took part in this study.

### Data analyses

Analyses were conducted at the word-level. Table 1 presents the average number of ratings per word, and the mean animacy ratings obtained from each sample. Following VanArsdall and Blunt’s criterion [1], we classified words into categories based on the average ratings obtained in each sample: inanimates (ratings ≤ 3), ambiguous (ratings between 3 and 5), and animates (ratings ≥ 5). We present the interrater agreement in terms of the words’ categorisation (Fleiss’s kappa, *κ*) and mean ratings (Intraclass Correlation Coefficient, ICC). The inter-age agreement considered the 224 and the 500 words rated by the Portuguese and the British samples, respectively; the comparisons between languages considered the 173 words shared by the pools of words used in each language. We also present mean-rating comparisons between age groups and within each language (paired t-tests) which allowed us to consider the maximum number of stimuli in the analyses. Then, we compared the mean ratings including Age and Language as variables in a repeated-measures ANOVA. Analyses were conducted using R [36] and SPSS 25 [37].

## Results

**Fig 2:**
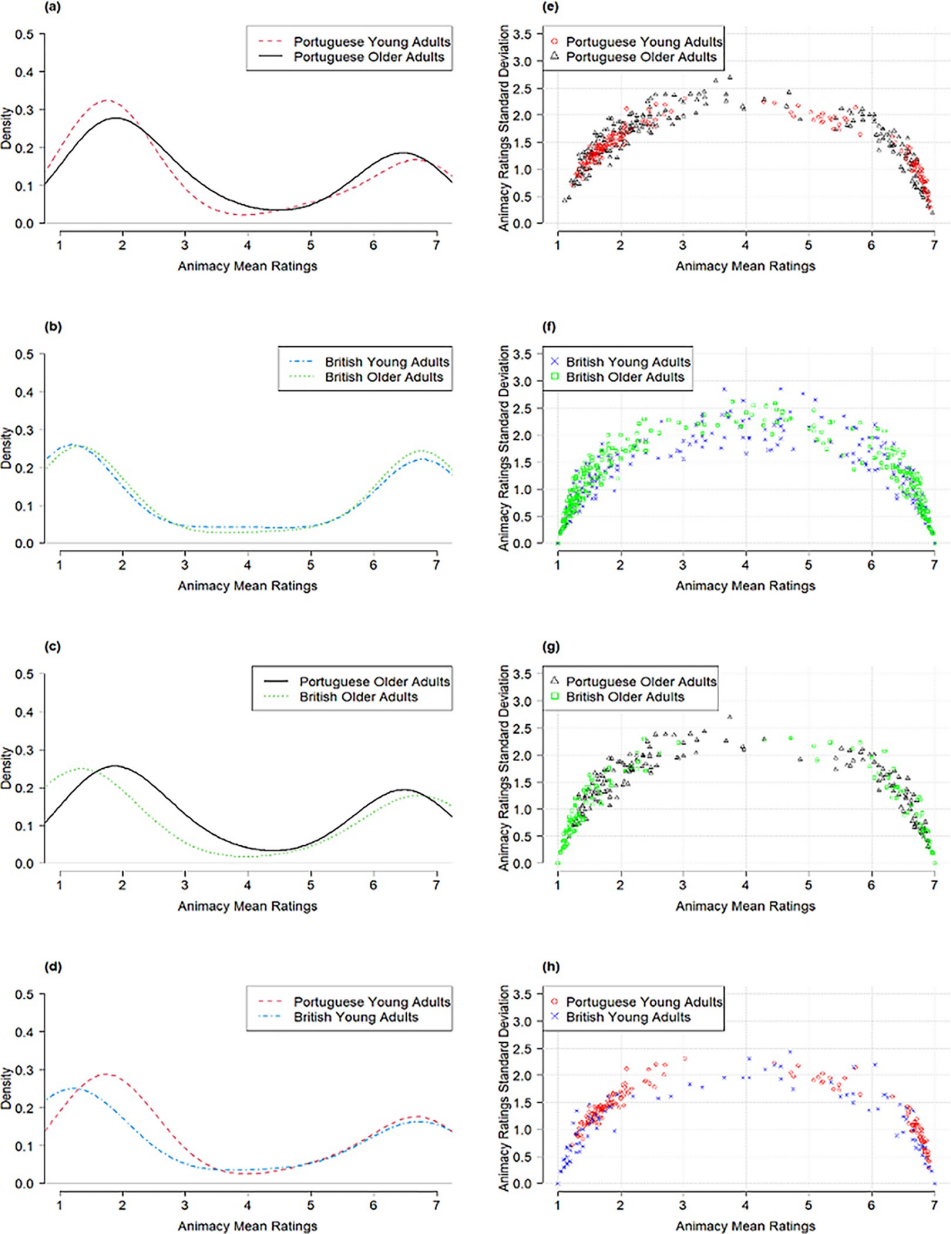
Distribution of Animacy Mean Ratings Across Age Groups and Languages (a-d), and as a Function of the Standard Deviations of the Ratings (e-h).

Table 2A–2D show the percentage of words categorised into animate, inanimate, and ambiguous across age groups and languages, along with the interrater agreements (both in terms of the words’ categorisation into animate/inanimate/ambiguous, *κ*; and the mean animacy ratings, ICC). Fig 2A–2D show the rating distributions provided by the different samples, whereas Fig 2E–2H plot the relationship between each word’s rating variability (*SD*) and its mean animacy rating.

**Table 2:** Percentage of words categorised into animates, inanimates and ambiguous by young and older adults, in European Portuguese and British English, and their interrater agreement. Overall Interrater Agreement, Intraclass Correlation Coefficient, and Pearson Correlations are also Presented.

(a)					Portuguese Young Adults									
					IN				AM				AN	
Portuguese Older Adults				IN	56.3				0.0				0.0	*κ* = .889, (95% CI, .758; 1.020), *p* < .001
				AM	5.4				1.8				0.4	*κ* = .313, (95% CI, .182; .443), *p* < .001
				AN	0.0				0.9				35.3	*κ* = .971, (95% CI, .840; 1.102), *p* < .001
Overall agreement					Animacy categorisation									*κ* = .871, (95% CI, .757; .986), *p* < .001
					Mean ratings									ICC = .988, (95% CI, .984; .990), *r* = .977, *p* < .001
(b)					British Young Adults									
					IN			AM				AN		
British Older Adults			IN		47.6			0.8				0.0		*κ* = .956, (95% CI, .868; 1.044), *p* < .001
			AM		1.2			3.8				0.0		*κ* = .546, (95% CI, .458; .633), *p* < .001
			AN		0.2			3.6				42.8		*κ* = .923, (95% CI, .835; 1.011), *p* < .001
Overall agreement					Animacy categorisation									*κ* = .896, (95% CI, .822; .971), *p* < .001
					Mean ratings									ICC = .992 (95% CI, .990; .993), *r* = .984, *p* < .001
(c)					British Older Adults									
					IN		AM				AN			
Portuguese Older Adults		IN			53.8		0.6				0.0			*κ* = .930, (95% CI, .781; 1.079), *p* < .001
		AM			2.3		0.6				2.9			*κ* = .137, (95% CI, -.012; .286), *p* = .072
		AN			0.6		0.0				39.3			*κ* = .928, (95% CI, .779; 1.077), *p* < .001
Overall agreement					Animacy categorisation									*κ* = .878, (95% CI, .743; 1.014), *p* < .001
					Mean ratings									ICC = .979, (95% CI, .972; .984), *r* = .971, *p* < .001
(d)					British Young Adults									
					IN	AM				AN				
Portuguese Young Adults	IN				54.9	3.5				0.0				*κ* = .917, (95% CI, .768; 1.067), *p* < .001
	AM				0.6	2.3				0.0				*κ* = .443, (95% CI, .294; .592), *p* < .001
	AN				0.0	1.2				37.6				*κ* = .976, (95% CI, .826; 1.125), *p* < .001
Overall agreement					Animacy categorisation									*κ* = .901, (95% CI, .771; 1.032), *p* < .001
					Mean ratings									ICC = .988, (95% CI, .983; .991), *r* = .981, *p* < .001

AM = Ambiguous words (3 < Mean ratings < 5); AN = Animate words (Mean ratings ≥ 5); IN = Inanimate words (Mean ratings ≤ 3).

### Age comparisons

Overall, there was a strong agreement between the young and older adults (in both languages) regarding the animacy categorisation. As Table 2A and 2B present, the strongest agreement was obtained when categorising words into animates and inanimates, and not so much for those classified as ambiguous [38, 39], a category that only contained a few words. Changes in the categorisation of words by the two age groups occurred for 6.7% of the Portuguese and 5.8% of the British words. In most cases, older adults categorised words into a higher animacy category than the young adults (Table 2A and 2B). Table 3 presents examples of those words.

**Table 3:** Examples of words categorised into a higher animacy category by the older than the young adults (Both in European Portuguese and British English).

	Categorised by		Examples of words
	Older Adults	Young Adults	
Portuguese	Animates	Ambiguous	*ear [orelha]*, *elbow [cotovelo]*
	Ambiguous	Inanimates	*hospital [hospital]*, *earth [terra]*, *sea [mar]*, *sky [céu]*, *lawn [relvado]*
British	Animates	Ambiguous	ear, elbow, nose, earth, garden, seed, plasma, blood, leg, tumour
	Ambiguous	Inanimates	village, apple, soil, almond, peanut, walnut
	Animates	Inanimates	egg

European Portuguese translations are presented in parenthesis.

The ICC values presented in Table 2A and 2B also denote a strong agreement on the mean ratings obtained for each word [40]. Nevertheless, the older adults provided, on average, higher animacy ratings than the young adults (Table 1), both in European Portuguese, *t*(223) = 5.19, *p* < .001, *dz* = 0.35, and in British English, *t*(499) = 7.77, *p* < .001, *dz* = 0.35. As depicted in Fig 2A and 2B, the young adults provided low animacy ratings (i.e., ratings below 3) more frequently than the older adults; the reverse occurred for the higher ratings (i.e., above 5). Fig 2E and 2F reveal a lower variability at the extremes of the scale (i.e., for words considered to be clearly animates/inanimates), and a higher variability towards the middle of the rating scale (i.e., ambiguous words). Additionally, the variability (as indexed by the *SD* obtained for each word) was significantly higher in the older than in the young adults, both in the Portuguese, *t*(223) = 4.83, *p* < .001, *dz* = 0.32 (Mean *SDs* obtained for each word: older adults = 1.48; young adults = 1.34), and the British samples, *t*(499) = 6.02, *p* < .001, *dz* = 0.27 (Mean *SDs* obtained for each word: older adults = 0.92; young adults = 0.75).

### Language comparisons

There was also a strong animacy agreement between languages (in both age groups; Table 2C and 2D), both when considering the mean ratings and the word categorisation based on the ratings of the 173 words shared among datasets. Still, on average, the Portuguese participants assigned higher animacy ratings (*M* = 3.74; *SD* = 2.28) than the British participants (*M* = 3.56; *SD* = 2.60), irrespectively of their age group [older adults: *t*(172) = 3.30, *p* = .001, *dz* = 0.25; young adults: *t*(172) = 4.50, *p* < .001, *dz* = 0.34]. This pattern is evident in the displacement of the curve to the right side in Fig 2C and 2D. Table 4 presents examples of such cases. Additionally, the variability (*SD*) in the ratings obtained for each word was significantly higher in the Portuguese than in the British samples, in both the older, *t*(172) = 13.07, *p* < .001, *dz* = 0.99, and the young adults, *t*(172) = 14.04, *p* < .001, *dz* = 1.07 (Fig 2G and 2H).

**Table 4:** Examples of words categorised into a higher animacy category by the European Portuguese than the British English samples.

	Categorised by		Examples of words
	Portuguese	British	
Older Adults	Animates	Inanimates	dinosaur
	Ambiguous	Inanimates	hospital, star, sky, river
Young Adults	Animates	Ambiguous	dinosaur, leg
	Ambiguous	Inanimates	egg

### Age X language comparisons

As before, these analyses relied on the ratings obtained for the 173 common words across samples. Their ratings were compared with a 2 (Age: Young vs. Older) x 2 (Language: European Portuguese vs. British English) repeated-measures ANOVA. The older adults assigned, on average, higher animacy ratings (*M* = 3.72; *SD* = 2.42) than the young adults (*M* = 3.57; *SD* = 2.47), *F*(1, 172) = 28.32, *MSE* = 3.87, *p* < .001, *η*_*p*_^*2*^ = .141. The Language main effect was also reliable, *F*(1, 172) = 19.07, *MSE* = 5.63, *p* < .001, *η*_*p*_^*2*^ = .100, revealing that the Portuguese samples gave, on average, higher animacy ratings than the British samples (*M* = 3.74, *SD* = 2.28; *M* = 3.56, *SD* = 2.60, respectively). The Age X Language interaction was non-significant, *F*(1, 172) < 1, *MSE* = 0.01, *p* = .816.

In response to the increasing concerns related to the psychological and neurological functioning of transgender and gender-diverse individuals [41], we re-ran all of the analyses excluding the British young adult participants who did not identify themselves as male or female (*n* = 5; corresponding to 3.1% of the sample; see Table 1). These analyses are presented in the S3 File. The results remained as just reported.

## Discussion

As denoted in the Introduction, the effect of animacy spreads to various cognitive processes, including memory performance [4, 8, 9], language [17], and attention [14–16]. However, evidence regarding this variable in older adults is scarce possibly due to the non-existence of animacy word-rating studies with such an age group, a gap we aim to fulfil with this work. Additionally, we collected data in two languages (European Portuguese and British English) and compared the ratings provided by older adults with those given by young adults. The results inform on cross-language and inter-age usability of animacy word ratings.

Our findings suggest a high agreement on the animacy judgments made by the Portuguese and British samples. Still, the Portuguese participants provided higher animacy mean ratings than the British participants, which may be due to cultural (e.g., [42]) and language/grammatical differences (e.g., the use of the English pronoun “it” to inanimates as well as to some animates, such as animals; whilst no similar pronoun exists in Portuguese). Previous studies have also reported that animacy ratings are consistent between languages [21, 23], even though speakers from different languages can process words differently [20]. For example, Serbian (comparatively to English) speakers took longer to process the meaning of inanimate words in a semantic categorisation task; however, the animacy word ratings of both groups were highly correlated [20].

We also report comparisons between age groups, which have not been done before and can be useful to future studies interested in exploring age differences. Animacy ratings of young and older adults were in high agreement, indicating that both age groups agree on whether a word is more animate or inanimate. However, the older adults tended to provide significantly higher animacy ratings than the young adults. Some of the words for which the rating difference was larger included *hospital*, *sea*, *earth*, and *blood*. This result highlights the importance of using age-specific word ratings when manipulating and/or controlling for animacy in (cognitive) research.

Age differences in the mean ratings of other semantic variables have been reported in other languages, despite the high interrater agreements (e.g., valence and arousal [28, 42]; imageability and emotionality [29]). Some authors reasoned that the age differences could be due to a higher language experience by older adults, compared to young adults [28, 29]. As such, for instance, the higher ability reported by older adults to mentally visualise words could result from that increased experience [29]. Additionally, those authors suggested that the older adults’ bias to more positive emotional ratings to specific words (e.g., “duty”, “chapel”, “god”) could reflect generational and societal changes [29].

The animacy age differences seem to be similar both in European Portuguese and British English as the Age X Language interaction was not significant. However, it is important to note that this analysis contained a relatively small number of words, and, thus, additional research is needed to fully support these conclusions. Also, the groups called upon were occidental, leaving open the question of the extent to which these age differences would be similar in other languages and/or cultures, as age patterns may be influenced by different cultural and/or historical environments [43]. Additionally, the meaning of words [44], as well as the implicit animacy-related grammatical rules (e.g., [23, 45]), are influenced by language and culture.

One could speculate about the causes underlying the age differences in the obtained animacy ratings. From a social point of view, the socioemotional selectivity theory predicts that, with ageing, the number of relationships reduces while their meaningfulness increases [46]. As such, older adults may focus on the implicit social aspects of a given concept more than young adults (e.g., *hospital*–doctors and nurses interacting with patients; *blood*–animals/humans need blood to stay alive). Sociocultural changes between the age groups may also underlie some of the differences in the mean ratings.

From a semantic network point of view, categorising things as non-living depends, to some extent, on accessing their functional features (e.g., *used to brush*), whereas the categorisation as living relies more on sensory/perceptual features (e.g., *has fur*). Older adults may tend to “misclassify” inanimates as animates (or, at least, assign them higher animacy ratings) because they tend to generate significantly more sensory features (typically more associated with animates) to non-living things than young adults [47]. This could also explain the higher animacy mean ratings provided by older (vs. young) adults.

Another interesting result obtained in our study refers to the higher rating variability observed in older adults as compared to young adults. These results could be due to differences in the semantic networks of young and older adults. The semantic representations (of animals, for instance) of both age groups, although similar in the clustering coefficients, also present some differences in connectivity, as they become sparser with aging [48]. This might result from the higher knowledge and experience with language of the older adults (vs. the young adults). The more idiosyncratic semantic networks likely occurring in older adults (vs. young adults) [48] may cause higher variability (*SDs*) in this age group’s ratings per word. We return to this point below.

These possible explanations are merely speculative as they are beyond the scope of this work. Nonetheless, our findings emphasise the need to consider animacy language- and age-specific word ratings in research using word materials.

The aim of this work was to make available the first set of animacy word ratings collected from older adults in European Portuguese and British English. Although we have accomplished this goal, some limitations can be pointed out to this work, and some considerations need to be made for future studies. First, even though we selected words that were quite familiar/frequent, future studies should include the response option "I do not know this word" to prevent random ratings in case participants are not familiar with specific words, and to identify such cases. Second, it would also be relevant to collect data on other individual variables that might influence the ratings in a general way, particularly when comparisons are to be drawn between young and older adults. These include variables such as years of education, general cognitive functioning, and fluid vs. crystallized intelligence. Even though we did not collect information on cognitive functioning, it has been reported that older adults with cognitive decline use computers less frequently [49], making it unlikely that such participants are widely represented in our sample. Regarding crystallized intelligence, which tends to be associated with more knowledge related to a longer lifelong experience, older adults usually score higher on this variable than young adults [29]. This reflects, for example, in fewer words rated as “unknown" [29] and in a smaller impact of the effect of words’ frequency in reading times [30] in older adults (vs. young adults). A higher crystallized intelligence also relates to the more extensive knowledge and experience with language which affords wider semantic networks on the older adults (as mentioned above), possibly affecting their word ratings [47, 48].

The participants’ sex is another variable that might influence word ratings. Previous work has revealed that males and females rate words differently, namely on emotion-related dimensions (e.g., [42, 50], but see [29]), but less evidence exists for other variables (e.g., imageability [29]). Given we were unable to balance our samples in terms of sex, we refrain to explore it in our data, although we make this available on our shared databases [31]. Thus, whether sex influences animacy ratings is also an open question for future research.

In the Introduction we mentioned that the correlations between animacy ratings and those of other semantic variables seem to be low [7, 8]. However, those relied on data collected from young adults. Exploring such relations with the older adults’ animacy ratings is challenging at this point as very few studies exist reporting data collected specifically from this age group (for recent exceptions see [28, 29, 51]), none of which with European Portuguese or British English participants. The fact that our results hint at age differences should inspire other work to also explore an influence of such variable in other semantic variables. This would, in turn, allow the investigation of relations among variables, animacy included.

All in all, the present animacy database constitutes an asset for researchers conducting studies with words, making it possible to control for and/or manipulate animacy in their work. The database is freely available through OFS [31].

## Supporting information

S1 File Characterisation of the rated words. S1 Table. Characterisation of the inanimate, ambiguous, and animate rated words.
(DOCX)

S2 File Animacy-rating instructions. (DOCX)

S3 File Additional analyses. S1 Table. Characterisation of the British Sample of Young Adults (N = 156), Mean Ratings per Word, and Average Animacy Ratings. S2 Table. Percentage of Words Categorised into Animates, Inanimates and Ambiguous by British Young and Older Adults, and British and European Portuguese Young Adults, and their Interrater Agreement. Overall Interrater Agreement, Intraclass Correlation Coefficient, and Pearson Correlations are also Presented.
(DOCX)
