# Supplementary material for: Is “earth” an animate thing? Cross-language and inter-age analyses of animacy word ratings in European Portuguese and British English young and older adults
Source: PLoS One. 2023 Aug 4;18(8):e0289755. doi: 10.1371/journal.pone.0289755 (PMC10403098; doi:10.1371/journal.pone.0289755)
Supplement: S1 File — S1 Table. Characterisation of the inanimate, ambiguous, and animate rated words. (DOCX) [file pone.0289755.s001.docx]

# Supporting information 1

## Characterisation of the rated words

**S1 Table.** **Characterisation of the Inanimate, Ambiguous, and Animate Rated Words.**

|  | European Portuguese Words (*N* = 224) | | |  | British English Words (*N* = 500) | | |
| --- | --- | --- | --- | --- | --- | --- | --- |
|  | IN  (*n* = 138) | AM  (*n* = 4) | AN  (*n* = 82) |  | IN  (*n* = 230) | AM  (*n* = 70) | AN  (*n* = 200) |
| ANIM^a,b^ | 1.80 (0.31) | 4.18 (0.84) | 6.35 (0.58) |  | 1.23 (0.28) | 4.03 (0.45) | 6.66 (0.36) |
| AoA^a,b^ | 2.77 (0.96) | 2.23 (0.74) | 2.89 (1.08) |  | 5.60 (1.67) | 6.51 (2.46) | 6.16 (1.90) |
| ARO^a,b^ | 4.28 (1.64) | 4.15 (2.37) | 4.47 (2.20) |  | 3.88 (0.90) | 3.87 (0.89) | 4.22 (0.96) |
| CNC^a,b^ | 6.31 (0.42) | 6.25 (0.68) | 6.18 (0.45) |  | 5.80 (0.46) | 5.80 (0.54) | 5.81 (0.53) |
| DOM^a,b^ | 5.15 (1.82) | 4.56 (2.48) | 5.12 (2.46) |  | 5.61 (0.73) | 5.40 (0.83) | 5.23 (0.86) |
| FAM ^b^ | --- | --- | --- |  | 5.50 (0.56) | 5.41 (0.63) | 5.51 (0.79) |
| IMG^a,b^ | 5.93 (0.40) | 5.88 (0.35) | 5.82 (0.46) |  | 5.80 (0.46) | 5.64 (0.85) | 5.78 (0.73) |
| LEN^a,b^ | 6.14 (1.78) | 5.25 (1.50) | 6.60 (2.17) |  | 5.59 (1.69) | 5.49 (1.47) | 5.90 (1.95) |
| NSyll^b,c^ | 2.69 (0.80) | 2.25 (0.50) | 2.78 (0.96) |  | 1.67 (0.75) | 1.66 (0.67) | 1.83 (0.77) |
| ON^b,c^ | 2.98 (3.87) | 2.00 (2.16) | 2.24 (3.34) |  | 5.43 (6.08) | 4.68 (5.74) | 5.00 (7.09) |
| PN^b,c^ | 3.20 (4.01) | 3.75 (3.30) | 2.40 (3.80) |  | 13.43 (14.05) | 12.98 (15.81) | 10.23 (13.09) |
| SF^a^ | 4.97 (1.15) | 4.99 (0.29) | 4.67 (1.06) |  | --- | --- | --- |
| VAL^a,b^ | 5.52 (2.16) | 4.87 (3.14) | 5.57 (2.82) |  | 5.71 (0.99) | 5.49 (1.21) | 5.68 (1.22) |
| WF^b,c^ | 74.33 (155.45) | 103.32 (139.99) | 72.20 (120.60) |  | 42.77 (66.14) | 18.67 (31.32) | 48.05 (99.98) |

Mean values are presented, with standard deviations in parenthesis.

The categorisation of the pool of words rated by the Portuguese sample was based on the ratings available in European Portuguese [1]; the categorisation of the English words was based on the data provided by American participants [2].

*N / n* - Number of words; AM - Ambiguous words (3 < Mean ratings < 5); AN - Animate words (Mean ratings ≥ 5); IN - Inanimate words (Mean ratings ≤ 3); ANIM - Animacy; AoA - Age of Acquisition; ARO - Arousal; CNC - Concreteness; DOM - Dominance; FAM - Familiarity; IMG - Imageability; LEN - Length; NSyll - Number of syllables; ON - Orthographic Neighbours; PN - Phonological Neighbours; SF - Subjective Frequency; VAL - Emotional Valence; WF - Written Frequency.

European Portuguese word data retrieved from: ^a^ [3] and ^c^ [4].

English word data retrieved from: ^b^ [2].

**References**

1. Félix SB, Pandeirada JNS, Nairne JS. Animacy norms for 224 European Portuguese concrete words. Análise Psicológica [Internet]. 2020;38:257–69. Available from: http://hdl.handle.net/10400.12/79

2. VanArsdall JE, Blunt JR. Analyzing the structure of animacy: Exploring relationships among six new animacy and 15 existing normative dimensions for 1,200 concrete nouns. Mem Cogn [Internet]. 2022;50:997–1012. Available from: https://doi.org/10.3758/s13421-021-01266-y

3. Félix SB, Pandeirada JNS. Norming studies of lexicosemantic and affective European Portuguese words: A literature review. Análise Psicológica [Internet]. 2021;39:107–31. Available from: http://hdl.handle.net/10400.12/8243

4. Soares AP, Comensaña M, Sanroman AI, Almeida JJ, Simões A, Costa A, et al. P-PAL: Uma base lexical com índices psicolinguísticos do Português Europeu. Linguamática [Internet]. 2010;2:67–72. Available from: https://linguamatica.com/index.php/linguamatica/article/view/80
