# Supplementary material for: Is “earth” an animate thing? Cross-language and inter-age analyses of animacy word ratings in European Portuguese and British English young and older adults
Source: PLoS One. 2023 Aug 4;18(8):e0289755. doi: 10.1371/journal.pone.0289755 (PMC10403098; doi:10.1371/journal.pone.0289755)
Supplement: S2 File — (DOCX) [file pone.0289755.s002.docx]

# Supporting information 2

## Animacy-rating instructions

### European Portuguese samples

*Based on the instructions used in Félix et al. [1]*

Tudo o que está em nosso redor pode ser um ser vivo, ou uma entidade não-viva. Nesta tarefa, pedimos que avalie algumas palavras quanto ao facto de se referirem a entidades vivas (animadas) ou não-vivas (inanimadas). A avaliação será feita numa escala de 1 a 7, em que 1 indica “totalmente inanimado / não-vivo” e 7 indica “totalmente animado / vivo”. As palavras que considere definitivamente animadas/vivas devem receber uma avaliação mais elevada na escala, enquanto que palavras inanimadas/não-vivas devem receber avaliações mais baixas.

Por exemplo: “Canguru” deve receber uma elevada avaliação de animacidade, dado referir-se a um animal (ser vivo). No entanto, a palavra “caneta” deve receber uma baixa avaliação de animacidade, dado referir-se a um objeto (entidade não-viva). Caso a palavra indique algo que não considera ser totalmente animado nem totalmente inanimado, deverá atribuir uma pontuação que se situe entre os extremos da escala.

As palavras apresentadas podem variar em muitas outras características. É importante que avalie as palavras somente quanto à animacidade, e não relativamente a quaisquer outras características.

Pode utilizar todos os valores da escala; não se deve preocupar se está a utilizar um determinado valor com maior frequência desde que este corresponda ao seu julgamento verdadeiro.

Não existem respostas certas ou erradas e não existe limite de tempo para cada resposta; pedimos, contudo, que responda de forma intuitiva, rápida e honesta.

### British English samples

*Based on the Living rating instructions from VanArsdall and Blunt [2]*

Things differ in whether they are living or nonliving. Some things may be very clearly living, whereas others may be very clearly nonliving. The purpose of this study is to rate a list of words with respect to the extent to which the thing represented by each word is living or nonliving.

Your ratings will be made on a seven-point scale, where "1" is the nonliving end of the scale and "7" is the living end of the scale. Make your rating by selecting the number from 1 to 7 that best indicates your judgment of whether the thing is living or nonliving.

Anything that you believe is definitely a living thing should be given a high living rating (at the upper end of the numerical scale). Anything that you believe is definitely a nonliving thing should be given a high nonliving rating (at the lower end of the numerical scale).

For example, the word “ballerina” should be given a high living rating, because ballerinas are people, and people are alive or living; thus, a rating of 7 could be assigned to this word. A word such as “piano”, on the other hand, should be given a high nonliving rating, because pianos are nonliving objects; thus, a rating of 1 could be attributed to this word.

Things that you believe are only mostly living or mostly nonliving should of course be rated appropriately between the two extremes. Feel free to use the entire range of numbers from 1 to 7 and also to use the end numbers of the scale.

Because words also differ in many other ways, it is important that your ratings not be based on these other characteristics and that you judge only how living or nonliving each thing is to you. Work fairly quickly, but do not be careless in your ratings.

## A condensed version of this scale will be presented at the top of each page for your reference.

**References**

1. Félix SB, Pandeirada JNS, Nairne JS. Animacy norms for 224 European Portuguese concrete words. Análise Psicológica [Internet]. 2020;38:257–69. Available from: http://hdl.handle.net/10400.12/79

2. VanArsdall JE, Blunt JR. Analyzing the structure of animacy: Exploring relationships among six new animacy and 15 existing normative dimensions for 1,200 concrete nouns. Mem Cogn [Internet]. 2022;50:997–1012. Available from: https://doi.org/10.3758/s13421-021-01266-y
