# Supplementary material for: Is “earth” an animate thing? Cross-language and inter-age analyses of animacy word ratings in European Portuguese and British English young and older adults
Source: PLoS One. 2023 Aug 4;18(8):e0289755. doi: 10.1371/journal.pone.0289755 (PMC10403098; doi:10.1371/journal.pone.0289755)
Supplement: S3 File — S1 Table. Characterisation of the British Sample of Young Adults (N = 156), Mean Ratings per Word, and Average Animacy Ratings. S2 Table. Percentage of Words Categorised into Animates, Inanimates and Ambiguous by British Young and Older Adults, and British and European Portuguese Young Adults, and their Interrater Agreement. Overall Interrater Agreement, Intraclass Correlation Coefficient, and Pearson Correlations are also Presented. (DOCX) [file pone.0289755.s003.docx]

# Supporting information 3

## Additional analyses

In response to the increasing concerns related to the psychological and neurological functioning of transgender and gender-diverse individuals [1], we re-ran the analyses excluding the participants who did not identify themselves as male or female (*n* = 5). This occurred only in the sample of British young adults, with 2.5% of the sample responding “other”, and 0.6% of the sample who preferred not to respond to such question. Below we present the sample characterisation and the obtained results (S1 Table) when these participants were excluded; this corresponds to a total sample of 156 British young adults. Here we report only the re-analyses that involve the data from this age group as the remaining analyses reported in the MS are unaffected. The pattern of obtained results was the same as that reported in the main manuscript (S2 Table).

**Age comparisons**

Concerning the British samples, the older adults provided, on average, higher animacy ratings than the young adults, *t*(499) = 7.69, *p* < .001, *dz* = 0.34. Also, the variability (as indexed by the *SD* obtained for each word) was significantly higher in the older than in the young adults, *t*(499) = 5.75, *p* < .001, *dz* = 0.26 (Mean *SDs* obtained for each word: older adults = 0.92; young adults = 0.76).

**Language comparisons**

The Portuguese participants assigned higher animacy ratings (*M* = 3.74; *SD* = 2.28) than the British participants (*M* = 3.89; *SD* = 2.61), irrespectively of their age group [young adults: *t*(172) = 4.43, *p* < .001, *dz* = 0.34].

Also, the variability (*SD*) in the ratings obtained for each word was significantly higher in the Portuguese than in the British samples [young adults, *t*(172) = 13.86, *p* < .001, *dz* = 1.05].

**S1 Table.** **Characterisation of the British Sample of Young Adults (N=156), Mean Ratings per Word, and Average Animacy Ratings.**

| Samples | %  Female/Male | Mean age  (*SD*; Range) | Mean ratings/word  (*SD*; Range) | Mean ratings (*SD*) |
| --- | --- | --- | --- | --- |
| British Young Adults | 46.2 / 53.8 | 27.8  (5.1; 18-35) | 19.50  (1.12; 17-21) | 3.80 (2.62) |

*SD* = Standard Deviation.

**S2 Table. Percentage of Words Categorised into Animates, Inanimates and Ambiguous by British Young and Older Adults, and British and European Portuguese Young Adults, and their Interrater Agreement. Overall Interrater Agreement, Intraclass Correlation Coefficient, and Pearson Correlations are also Presented.**

|  |  | British Young Adults | | |  |
| --- | --- | --- | --- | --- | --- |
|  |  | IN | AM | AN |  |
| British Older Adults | IN | 47.4 | 1.0 | 0.0 | *κ* = .952, (95% CI, .868; 1.044), *p* < .001 |
|  | AM | 1.2 | 3.8 | 0.0 | *κ* = .536, (95% CI, .448; .624), *p* < .001 |
|  | AN | 0.2 | 3.6 | 42.8 | *κ* = .923, (95% CI, .835; 1.011), *p* < .001 |
| Overall agreement | | Animacy categorisation | | | *κ* = .893, (95% CI, .818; .967), *p* < .001 |
|  |  | Mean ratings | | | ICC = .992 (95% CI, .990; .993), *r* = .984, *p* < .001 |

|  |  | British Young Adults | | |  |
| --- | --- | --- | --- | --- | --- |
|  |  | IN | AM | AN |  |
| Portuguese Young Adults | IN | 54.9 | 3.5 | 0.0 | *κ* = .917, (95% CI, .768; 1.067), *p* < .001 |
|  | AM | 0.6 | 2.3 | 0.0 | *κ* = .443, (95% CI, .294; .592), *p* < .001 |
|  | AN | 0.0 | 1.2 | 37.6 | *κ* = .976, (95% CI, .826; 1.125), *p* < .001 |
| Overall agreement | | Animacy categorisation | | | *κ* = .901, (95% CI, .771; 1.032), *p* < .001 |
|  |  | Mean ratings | | | ICC = .988, (95% CI, .983; .991), *r* = .981, *p* < .001 |

AM = Ambiguous words (3 < Mean ratings < 5); AN = Animate words (Mean ratings ≥ 5); IN = Inanimate words (Mean ratings ≤ 3).

## Age X Language comparisons

Analyses conducted with the ratings of the 173 common words across samples. A 2 (Age: Young vs. Older) x 2 (Language: European Portuguese vs. British English) repeated-measures ANOVA was conducted. The older adults assigned, on average, higher animacy ratings (*M* = 3.72; *SD* = 2.42) than the young adults (*M* = 3.58; *SD* = 2.47), *F*(1, 172) = 27.77, *MSE* = 3.84, *p* < .001, *η_p_^2^* = .139. The Language main effect was also reliable, *F*(1, 172) = 18.85, *MSE* = 5.58, *p* < .001, *η_p_^2^* = .099, revealing that the Portuguese samples gave, on average, higher animacy ratings than the British samples (*M* = 3.74, *SD* = 2.28; *M* = 3.56, *SD* = 2.60, respectively). The Age X Language interaction was non-significant, *F*(1, 172) < 1, *MSE* < 0.01, *p* = .842.

**Reference**

1. Warrier V, Greenberg DM, Weir E, Buckingham C, Smith P, Lai M-C, et al. Elevated rates of autism, other neurodevelopmental and psychiatric diagnoses, and autistic traits in transgender and gender-diverse individuals. Nat Commun [Internet]. 2020;11:3959. Available from: http://dx.doi.org/10.1038/s41467-020-17794-1
